# Explore the attitudes of children and adolescent parents towards the vaccination of COVID-19 in China

**DOI:** 10.1186/s13052-022-01321-7

**Published:** 2022-07-23

**Authors:** Lin Wang, Wen Wen, Chen Chen, Jiake Tang, Chunyi Wang, Mengyun Zhou, Yongran Cheng, Xingwei Zhang, Mingwei Wang, Zhanhui Feng, Weiqian Wang

**Affiliations:** 1 Hangzhou Dental Hospital, Hangzhou, 310063 China; 2 Hangzhou Institute of Cardiovascular Diseases,Affiliated Hospital of Hangzhou Normal University, Hangzhou, 310015 China; 3 Hangzhou Normal University, Hangzhou, 311121 China; 4 Shinshu University Graduate School of Medicine School of Medicine, Shinshu Daigaku Daigakuin Igakukei Kenkyuka Igakubu, Matsumoto, 3900803 Japan; 5 School of Public Health, Hangzhou Medical College, Hangzhou, 311300 China; 6 Department of Neurology, Affiliated Hospital of Guizhou Medical University, Guiyang, 550004 China

**Keywords:** COVID-19 vaccine, Vaccine hesitancy, Children, Adolescents, Parents

## Abstract

**Background:**

With the increasing incidence of asymptomatic carriers or milder symptoms, children and adolescents are likely to become a silent source of infection. In view of the efficacy and safety of vaccines in the treatment of novel Coronavirus pneumonia, population-wide vaccination will be an inevitable trend to control the spread of COVID-19. However, there is no survey on the attitudes of Chinese parents of children and adolescents towards their children’s COVID-19 vaccination.

**Methods:**

We used online questionnaires to find out the attitudes of Chinese parents toward their children’s immunization against COVID-19. Logistic regression was used to explore the influencing factors.

**Results:**

A total of 2019 parents participated in the survey. Overall, 74.38% parents said they would actively get vaccinated, 8.90% refused to get vaccinated, 4.60% said they would delay vaccination and 12.12% were still undecided.

**Conclusions:**

In general, Chinese parents have a high desire to be vaccinated against COVID-19, and most parents have a positive attitude towards their children’s vaccination. However, many people still hesitate or even refuse to be vaccinated. Education background, attitudes towards children’s vaccination, children’s age, recent illness and other factors have a certain impact on Chinese parents of children and adolescents towards their children’s COVID-19 vaccination.

**Supplementary Information:**

The online version contains supplementary material available at 10.1186/s13052-022-01321-7.

## Introduction

Novel Coronavirus infections pose a serious threat to the health and safety of the public, with over 260 million COVID-19 cases and over 5.21 million deaths worldwide as of December 1, 2021 [1]. COVID-19 has been mostly seen in adults since the outbreak, and children consistently accounted for a small proportion of the total number of coronavirus disease cases in 2019, with pediatric cases accounting for only 2% of the 80,900 COVID-19 cases during the surge in China [2, 3]. Although the majority of pediatric cases of COVID-19 are asymptomatic or mild, with a good prognosis and low mortality, novel coronavirus infection of severe acute respiratory syndrome (SARS) has been reported in children of all age groups [2, 4]. In addition, the disease may progress to multisystem inflammatory syndrome (MTIS), a life-threatening complication of COVID-19 infection [5]. Results of MTIS include myocardial dysfunction, shock, and respiratory failure and require intensive care. Study indicated that 18.4 out of 100,000 children aged 0–4 years and 10.6 out of 100,000 children aged 5–17 years required hospitalization, of which one third required intensive care.

There has recently been increasing evidence that people who are fully vaccinated with the mRNAs (BNT162b2 or mrna-1273) are less likely to develop symptomatic infections and transmit SARS coronavirus 2 (novel coronavirus) to others than those who are not vaccinated [5, 6]. Vaccines tested in young adults over the age of 12 were reported to be safe and effective, including mRNA vaccines produced by Moderna and Pfizer-Biontech [7, 8]. In addition, phase I/II clinical trial results of two inactivated Chinese vaccines produced by Sinovac and Sinopharm showed that the vaccines were well tolerated, safe and could induce humoral reactivity in children and adolescents aged 3–17 years [9]. While vaccinating children and adolescents is seen as key to ending the epidemic, Vaccine Hesitancy (VH) -- the delayed acceptance or refusal of a vaccine despite its availability -- remains a barrier to full population vaccination [5, 10]. Alfieri et al.’s study showed that 33% of parents were hesitant to vaccinate their children against COVID-19 [11]. Separate data from the US showed that 33% of parents said it was very unlikely that their children would be vaccinated against COVID-19 and 12% said they were not sure [12].

As the incidence of asymptomatic carriers is getting higher and higher, the symptoms of patients with COVID-19 infection are getting lighter and lighter, children and adolescents are likely to become a silent source of infection and remain a threat, becoming an important source of sustained transmission [5, 13]. In order for vaccines to be effective in controlling the spread of COVID-19, it is estimated that 67% of the population needs to be vaccinated to achieve herd immunity [11, 14], and a comprehensive push to vaccinate adults, children and adolescents is an inevitable trend in controlling the spread of COVID-19. In addition, for children under the age of 18, such as parents’ behaviors and values tend to have a great impact on their children. This paper mainly explores parents’ attitudes towards COVID-19 vaccination and related influencing factors, so as to provide corresponding guidance for popularizing vaccination for children and adolescents.

## Method

### Population and sampling

**Fig. 1:**
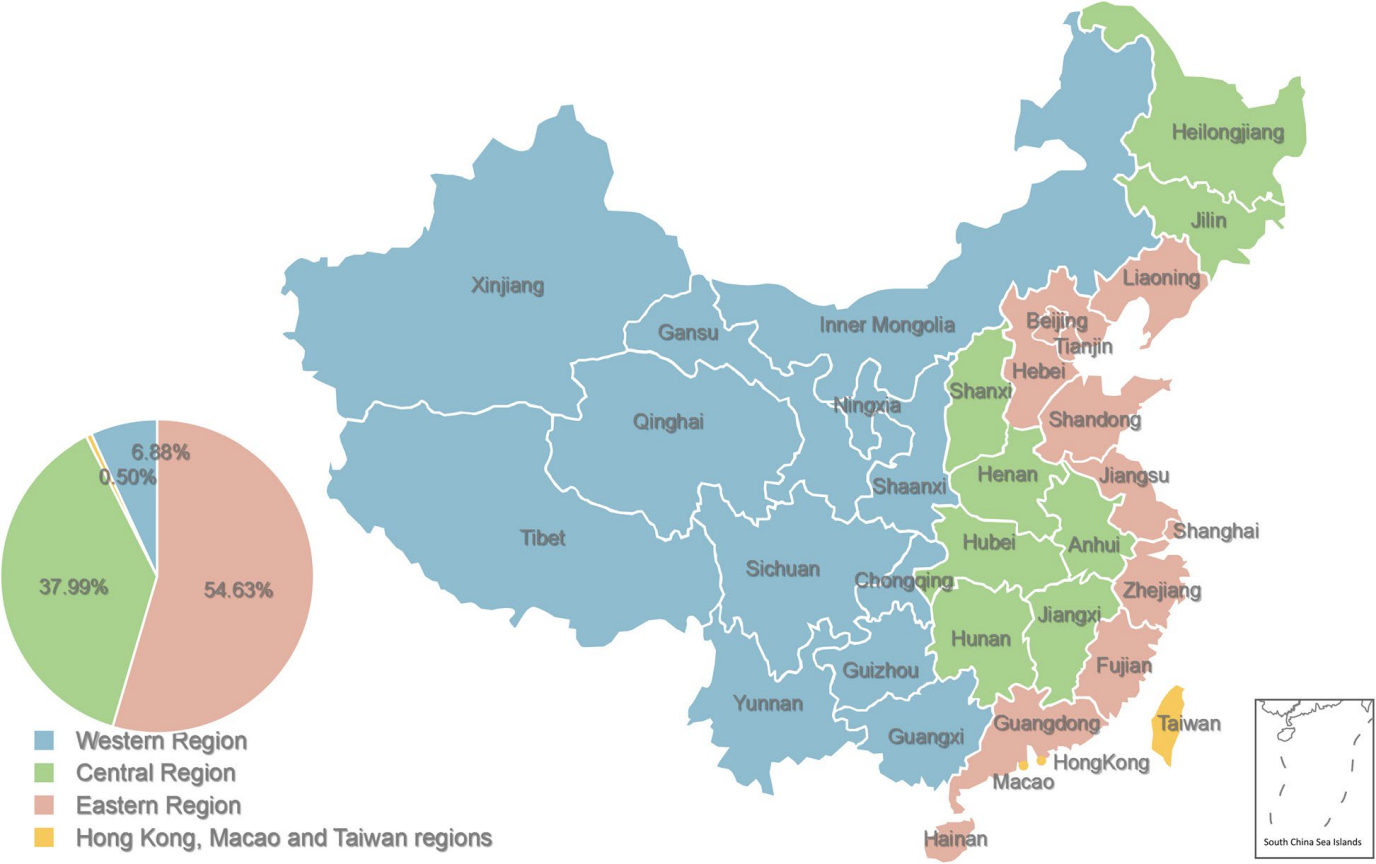
The respondents of our questionnaire came from Eastern Region, Central Region, Western Region, Hong Kong region, Macau region and Taiwan region, China

This study was a nation-wide cross-sectional study in China (Fig. 1). The ethics committee of Hangzhou Stomatological Hospital approved all the procedures performed. In November 2021, an anonymous online cross-sectional survey was conducted on Wen Juan network (https://www.wenjuan.com), set by Shanghai Zhongyan Network Technology. It is the largest free online survey platform in China, which can provide questionnaire creation, release, management, collection, and analysis services for enterprises or individuals. Their personal information can be confirmed,authentic,diverse and representative samples can be obtained. Participants are parents of Chinese children and adolescents (< 18 years old). Questionnaires were filled anonymously and 2019 copies were included apart from 131 invalid and wrong questionnaires.

### Questionnaire design

Informed consent had been designed. We set that if the respondent agrees to answer, they can continue to complete the questionnaire. Otherwise they cannot continue.

This questionnaire involved the following aspects (Supplementary File 1):

the twelve basic personal information such as gender, age, family role, level of education, habitual residence, annual household income, the number, gender and age of children, insurance coverage for the children, whether a single parent family and household health care workers or not; and other five aspects of the opinions about the vaccine, for example, “Have you been vaccinated against COVID-19”, “Have your children had a history of other diseases in the last 3 months”, “Have you explained the COVID-19 vaccine to your children”,“What’s your attitude towards children’s vaccination”,“Do you think there are vaccine side effects”. The explanation for the COVID-19 vaccin is for children over the age of 3.

### Statistical method

A descriptive statistical analysis was conducted on the social and demographic characteristics of the sample, and the corresponding proportion was calculated accordingly. Parental acceptability of COVID-19 vaccination was used as the dependent variable. A univariate logistic regression model first assessed the significance of the association between each of the background characteristics and the dependent variable. All relationships between the predictor and criterion variables were represented as odds ratios (OR) with 95% confidence intervals. The statistical tests were two-sided, and the effects with *p* < 0.05 were considered to be statistically significant. All statistical models were constructed using R software version 3.6.0 (R Foundation for Statistical Computing, version 3.6.1; http://www.Rproject.org).

## Results

### Demographic information

A total of 2019 valid questionnaires, and the basic information of the respondents was summarized in Table 1. Among the respondents, 738 (36.6%) were males, 1281 (63.4%) were females, and 422 (20.9%) were under the age of 30 years. 41.2% had a junior high school degree, 30.4% had a bachelor’s degree, 21.6% had a junior college degree, and the rest 6.8% had a master’s degree or above. In terms of place of residence, 10 parents came from Hong Kong, Macao and Taiwan, 1103 from central China, 139 from western China and 767 from eastern China. As for the annual household income, 26.7% of respondents reported less than 20,000 RMB per year, 22.3% between 20,000 and 50,000 RMB, 31.3% between 50,000 and 150,000 RMB, and 19.7% over 150,000 RMB. A total of 1501 parents of children and adolescents said they would be willing to be vaccinated against COVID-19 and another 518 said they would not. More than 80% of the parents had explained information about COVID-19 vaccine to their children, and more than 90% of the respondents had received COVID-19 vaccine therapy. Most (54.0%) of the children were aged 3–8, and 57.3% of them were boys. Furthermore, most children had health insurance (93.1%), with 10% having commercial insurance, 67.7% having medical insurance and 15.4% having both types of insurances.

**Table 1:** Baseline data

	Unwillingness(*N* = 518)	Willingness(*N* = 1501)	*p* value
Your role in the family			0.449
Father	197 (38.0)	541 (36.0)	
Mother	321 (62.0)	960 (64.0)	
Your age			0.302
< 30 years old	117 (22.6)	305 (20.3)	
≥ 30 years old	401 (77.4)	1196 (79.7)	
Has your child had a history of other diseases in the last 3 months			< 0.001
Yes	317 (61.2)	417 (31.4)	
No	201 (38.8)	1084 (72.6)	
Habitual residence			0.627
Hong Kong Macao and Taiwan	4 (0.8)	6 (0.4)	
Central China	290 (56.0)	813 (54.2)	
Western China	35 (6.8)	104 (6.9)	
Eastern China	189 (45.9)	578 (38.5)	
The gender of your child			0.098
Boy	280 (54.1)	876 (58.4)	
Girl	238 (45.9)	625 (41.6)	
The age of your child			< 0.001
0–2 year	105 (20.3)	163 (10.9)	
3–8 year	291 (56.2)	800 (53.3)	
9–12 year	85 (16.4)	237 (15.8)	
13–17 year	37 (7.1)	301 (20.1)	
Number of children in your family			0.23
One	282 (54.4)	831 (55.4)	
Two	215 (41.5)	583 (38.8)	
Three or more	21 (4.1)	87 (5.8)	
Your attitude towards children’s vaccination			< 0.001
Active	191 (36.9)	1428 (95.1)	
Refuse	64 (12.4)	4 (0.3)	
Delay	85 (16.4)	15 (1.0)	
Hesitate	178 (34.4)	54 (3.6)	
Do you think there are vaccine side effects?			< 0.001
Yes	331 (63.9)	715 (47.6)	
No	187 (36.1)	786 (52.4)	
Insurance coverage for your children			< 0.001
Commercial insurance	74 (14.3)	66 (4.4)	
Both commercial and health insurance	84 (16.2)	227 (15.1)	
None	61 (11.8)	141 (9.4)	
Health insurance	299 (57.7)	1067 (71.1)	
Annual household income			< 0.001
0–20,000	94 (18.1)	446 (29.7)	
20,000–50,000	100 (19.3)	350 (23.3)	
50,000–150,000	175 (33.8)	456 (30.4)	
150,000–500,000	118 (22.8)	201 (13.4)	
> 500,000	31 (6.0)	48 (3.2)	
Your level of education			0.027
Junior-senior high school	202 (39.0)	630 (42.0)	
Undergraduate college	163 (31.5)	451 (30.0)	
Graduate or above	49 (9.5)	88 (5.9)	
junior college	104 (20.1)	332 (22.1)	
Have you been vaccinated against COVID-19?			< 0.001
No	123 (23.7)	50 (3.3)	
Yes	395 (76.3)	1451 (96.7)	
Whether it is a single parent family			0.872
No	338 (65.3)	1021 (68.0)	
Yes	180 (34.7)	480 (32.0)	
Whether members of the household have health care workers?			0.290
Yes	251 (48.5)	685 (45.6)	
No	267 (51.5)	816 (54.4)	

### Related factors affecting parents’ willingness to vaccinate their children against COVID-19

Several factors were taken into consideration as for parents’ wilingness (Table 2). Gender, age, place of residence, number of children in the family, gender of children, whether a single parent family or not, and whether there was a doctor in the family had no statistical significances(*p* > 0.05), indicating that these factors had no significant impact on parents’ willingness to vaccinate their children against COVID-19. The children with no history of other diseases (such as cold, fever, diarrhea, etc.) were more likely to be vaccinated than those with other diseases in the last 3 months (OR = 1.29;95%CI 1.24–1.36, *p* < 0.001). We analyzed parents’ perceptions of their children’s vaccinations. Research showed children and adolescents were less likely to be vaccinated when their parents showed refusal (OR = 0.03;95% CI 0.01–0.04), delay (OR = 0.02; 95% CI 0.01–0.04) and hesitation (OR = 0.04;95% CI 0.03–0.06) respectively comparing with children whose parents maintained positive attitudes. Parents’ perceptions of vaccine side effects also influenced children’s willingness to be vaccinated, with parents feeling that vaccines had no side effects, children were more likely to be vaccinated (OR = 1.51; 95% CI 1.42–1.73, *p* < 0.001). Besides, when parents were vaccinated against COVID-19, their children were more likely to be vaccinated (OR = 3.29; 95% CI 1.10–9.85, *p* = 0.033). Compared with uninsured children, insured children were more likely to be vaccinated, especially those with health insurances (OR = 4.00; 95% CI 2.80–5.71, *p* < 0.001). Children of parents with graduate education or above were also more likely to be vaccinated (OR = 0.58; 95% CI 0.39–0.85, *p* = 0.005).

**Table 2:** Analysis of influence factors

	OR	95%CI	p值
Your role in the family			
Father	ref		
Mother	1.09	0.89–1.34	0.419
Your age			
< 30 years old	ref		
≥ 30 years old	1.14	0.9–1.46	0.277
Has your child had a history of other diseases in the last 3 months			
Yes	ref		
No	1.29	1.24–1.36	< 0.001
Habitual residence			
Hong Kong Macao and Taiwan	ref		
Central China	1.87	0.52–6.67	0.335
Western China	1.98	0.53–7.43	0.311
Eastern China	2.04	0.57–7.3	0.274
The gender of your child			
Boy	ref		
Girl	0.84	0.69–1.03	0.088
The age of your child			
0–2 year	ref		
3–8 year	1.77	1.34–2.34	< 0.001
9–12 year	1.80	1.27–2.55	< 0.001
13–17 year	5.24	3.44–7.98	< 0.001
Have you explained the COVID-19 vaccine to your child? (> 3 years old)			
Yes	ref		
No	5.26	4.16–6.65	< 0.001
Number of children in your family			
One	ref		
Two	1.53	0.93–2.52	0.09
Three or more	1.09	0.88–1.34	0.43
Your attitude towards children’s vaccination			
Active	ref		
Refuse	0.03	0.01–0.04	< 0.001
Delay	0.02	0.01–0.04	< 0.001
Hesitate	0.04	0.03–0.06	< 0.001
Do you think there are vaccine side effects?			
Yes	ref		
No	1.51	1.42–1.73	< 0.001
Insurance coverage for your children			
Commercial insurance	ref		
Both commercial and health insurance	3.03	2.00–4.59	< 0.001
None	2.59	1.66–4.05	< 0.001
Health insurance	4.00	2.80–5.71	< 0.001
Annual household income			
0–20,000	ref		
20,000–50,000	0.74	0.54–1.01	0.058
50,000–150,000	0.55	0.41–0.73	< 0.001
150,000–500,000	0.36	0.26–0.49	< 0.001
> 500,000	0.33	0.20–0.54	< 0.001
Your level of education			
Junior-senior high school	ref		
Undergraduate college	0.89	0.7–1.13	0.326
Graduate or above	0.58	0.39–0.85	0.005
junior college	1.02	0.78–1.34	0.866
Have you been vaccinated against COVID-19?			
No	ref		
Yes	3.29	1.10–9.85	0.033
Whether it is a single parent family			
No	ref		
Yes	1.02	0.83–1.27	0.829
Whether members of the household have health care workers?			
Yes	ref		
No	0.89	0.73–1.09	0.267

### The regional distribution of parents’ willingness to vaccinate their children against COVID-19

**Fig. 2:**
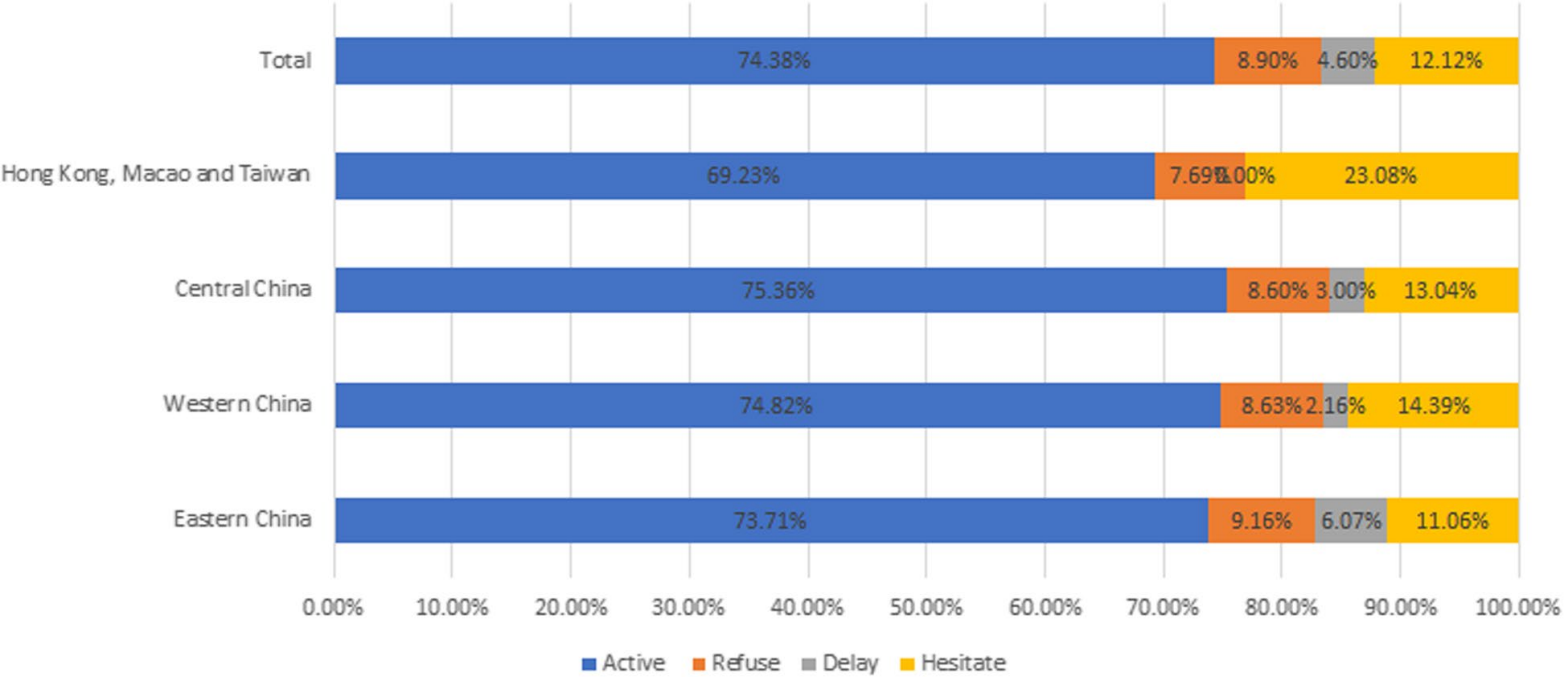
The regional distribution of parents’s willingness to be vaccinated against COVID-19

Figure 2 depicted the regional distribution of parents’ willingness to be vaccinated against COVID-19. Overall, 74.38% parents said they would actively get vaccinated for children or adolescents, 8.90% refused to get vaccinated, 4.60% said they would delay vaccination and 12.12% were still undecided. Our results showed that the willingness of parents in central China to be vaccinated was more than 75%. While the hesitation rate of parents in Hong Kong, Macao and Taiwan were 23.08%.

## Discussion

This study contributed to a better understanding of parents’ attitudes towards their children’s COVID-19 vaccination. In this study, 74.38% would actively get vaccinated. Overall, Chinese parents have high willingness to vaccinate children and adolescents against COVID-19. In addition, the study found that 80.19% of parents said they would actively vaccinate their children against COVID-19, 3.37% refused to vaccinate their children, 4.95% said they would delay vaccination, and 11.49% were still hesitant. More and more evidences reveal that vaccination against COVID-19 is currently an important tool to reduce the burden of the COVID-19 epidemic. Rhodes et al. showed parents scored below the midpoint on the willingness to vaccinate their children (M = 3.55, SD = 2.13) [15]. Our research showed that nearly 15% of parents were still refusing to vaccinate their children or are hesitant to do so. Our findings are similar to the survey study from Turkey [16]. Therefore, it is necessary to conduct our questionnaire to encourage and publicize the importance of COVID-19 vaccination, so as to control the COVID-19 epidemic earlier in the world.

There is growing evidence that genetic and viral vector COVID-19 vaccines prevent not only severe disease but also asymptomatic infection [17]. And a study from the United Kingdom found that adults infected 3 weeks after receiving a dose of Pfizer-Biotech or Astrazeneca vaccine were 38 to 49% less likely to pass the virus on to their family contacts than unvaccinated individuals [18]. However, trust in vaccines is still a significant factor influencing vaccination willingness. The main reasons cited by the study for parents’ reluctance to vaccinate their children against COVID-19 included a lack of adequate scientific research (84.8%), concerns about safety and side effects (76.9%), and potential inefficacy of the vaccine due to mutations (36.7%)5. Our research suggests that parents believe the COVID-19 vaccine has no side effects, and that children are more likely to be vaccinated (OR = 1.51; 95% CI 1.42–1.73, *p* < 0.001). Therefore, access to more positive information about the safety and effectiveness of adolescent COVID-19 vaccines, as well as school COVID-19 vaccination requirements, are the most frequently reported factors that increase the willingness of parents and adolescents to be vaccinated [19, 20]. In addition, Marquez et al. found that 39.2% of parents refused to vaccinate their children against COVID-19, while 27.8% agreed that they would allow their children to be vaccinated against COVID-19 if their doctor recommended it20. Health workers’ advocacy of COVID-19 vaccines is one of the most important sources of reliable vaccine information for the public. Increased clear public communication about the benefits and safety of COVID-19 vaccines for children and adolescents, especially by healthcare professionals, will help increase parents’ confidence in COVID-19 vaccines and their willingness to vaccinate their children against COVID-19. Our result showed their children were more likely to be vaccinated when parents were vaccinated against COVID-19. It’s very critical to educate clear information and transparent communication from public health, governments and leaders about the safety of childhood COVID-19 vaccines [12, 21].

We analyzed the factors that may influence children’s willingness to be vaccinated against COVID-19 and found that gender, age, place of residence, number of children in the household, gender of children, whether it’s a single parent family and whether there is a doctor in the household had no significant influences on the willingness of children and adolescents to be vaccinated against COVID-19. Children who did not get sick were more likely to get vaccinated than those who had other illnesses in the last 3 months. Different from the previous studies, which showed that younger adolescents were more likely to receive COVID-19 vaccination than older adolescents [22], while our study found that older children (13–17 years old) were more likely to be vaccinated against COVID-19. This may be due to China’s implementation of the COVID-19 vaccination program by age, which partially affected the results of the study. Lack of knowledge about COVID-19 and vaccines may increase misunderstandings about COVID-19 vaccines, thereby reduce the willingness of parents to vaccinate children and adolescents.

Parents do have significant effects on their children. On the one hand, studies have found that children and adolescents are less likely to get vaccinated when their parents show signs of rejection, delay, and hesitation. Children were more likely to be vaccinated if their parents felt there were no side effects; and when their parents have been vaccinated against COVID-19, children are more likely to be vaccinated. Our study suggests that parents play a non-negligible and even decisive role in the vaccination of children and adolescents against COVID-19. Parents’ attitudes towards vaccination and their views on vaccines will affect their children’s vaccination willingness. On the other hand, research from Italy shows the lower the education level of the parents, the greater the harm they think the COVID vaccine will do to their children [23]. Our result showed children of parents with graduate education or above were more likely to be vaccinated too. Therefore, it is necessary to raise parents’ awareness of the effectiveness and safety of the vaccine, so as to increase the COVID-19 vaccination rate in children and adolescents.

There did exist some shortcomings in our study. Firstly, we adopted the form of online questionnaire, which was mainly written by parents and indirectly investigated the vaccination intention of children and adolescents. There may be some errors in the data and relevant information to a certain extent. Secondly, the sample size of Hong Kong, Macao and Taiwan was too small,so it’s very necessary to gather more different regions to participate the survey.

## Conclusion

In general, Chinese children and adolescents have high desires to be vaccinated against COVID-19, and most parents have positive attitudes towards their children’s vaccination. However, many people still hesitate or even refuse to be vaccinated. From the study, we find that parents’ COVID-19 vaccine behaviors, education backgrounds, attitudes towards children’s vaccination, children’s age, recent illness and other factors have certain impacts on children and adolescents’ willingness to vaccinate against COVID-19.

## Supplementary Information

**Additional file 1: Supplementary file 1.** Original questionnaire data.

## Data Availability

Availability of data and material at corresponding author.

## Abbreviations

COVID-19: Coronavirus Disease 2019
SARS: Severe Acute Respiratory Syndrome
MTIS: Multisystem Inflammatory Syndrome
